# Supporting the involvement of older adults with complex needs in evaluation of outcomes in long‐term care at home programmes

**DOI:** 10.1111/hex.13484

**Published:** 2022-04-19

**Authors:** Lyn Phillipson, Ann‐Marie Towers, James Caiels, Louisa Smith

**Affiliations:** ^1^ School of Health and Society, Faculty of Arts, Social Sciences and Humanities and Australian Health Services Research Institute University of Wollongong Wollongong New South Wales Australia; ^2^ School for Social Policy, Sociology and Social Research, Reader in Social Care, Centre for Health Services Studies (CHSS) University of Kent Canterbury UK; ^3^ Personal Social Services Research Unit (PSSRU) University of Kent Canterbury UK; ^4^ Australian Health Services Research Institute University of Wollongong Wollongong New South Wales Australia

**Keywords:** ageing, cognitive impairment, dementia, evaluation, home care, long term care, quality of life

## Abstract

**Background:**

It is important to involve older people in evaluating public programmes that affect their lives. This includes those with physical and cognitive impairments (such as dementia) who may need support to live at home. Many countries have implemented new approaches to support older people to live well at home for longer. However, it can be challenging to involve disabled people in service evaluation, so we are unclear whether services are meeting their needs.

**Aim:**

This study explored how a cascading methodology, offering different supports enabled the involvement of home care users with cognitive and physical impairments in the assessment of their care‐related quality of life.

**Method:**

We used multiple tools from the Adult Social Care Outcomes Toolkit (ASCOT) with *n* = 63 older adults who were recipients of home care in the Illawarra. We also offered different physical and cognitive supports as needed.

**Results:**

We started with the standard ASCOT questionnaire to assess the care‐related quality of life, but then offered alternative formats (including Easy Read) and supports (including physical and cognitive assistance) if the older person needed them to participate. This allowed us to involve a greater diversity of older people in the evaluation, and changed what we found out about whether their care needs were being met.

**Conclusion:**

There is a need to implement more flexible and inclusive methods to increase the involvement of vulnerable users of long‐term care in the assessment of service outcomes. This is important to ensure that the perspectives of all service users inform the delivery of person‐centred care. It is also critical to understand the extent to which programmes are meeting the needs of vulnerable service users.

**Patient or Public Contribution:**

Service users with dementia were involved in the design of the ‘Easy Read’ questionnaire used in the study.

## 1 INTRODUCTION

With the global ageing of the population it is becoming increasingly important that we find ways to engage and involve all older people in policymaking and evaluating public programmes that affect their lives.^1^ However, involvement and engagement can present challenges with large numbers of older people living with chronic and degenerative conditions including dementia.^1^, ^2^

One key area that could benefit from greater patient involvement is the evaluation of aged care policies and programmes. Of particular need are those that emphasize ageing at home, with long‐term care services delivered in community settings.^3^, ^4^ Reforms in aged and disability care in many countries have supported the introduction of consumer‐directed care (CDC) models on the basis that they can improve autonomy and choice for service users.^5^, ^6^, ^7^, ^8^ However, there is mixed evidence concerning the effectiveness of these models,^8^, ^9^, ^10^ especially with regard to supporting vulnerable service users, such as those with dementia, mental health issues, financial or social disadvantage and/or low literacy.^6^, ^7^, ^11^, ^12^, ^13^, ^14^

One reason that we lack clear evidence about the effectiveness of different models of care in this group is the failure to use inclusive research methods to evaluate service‐related outcomes. Service evaluation from the patient perspective is especially complex given the vulnerability and diversity of impairments of users of aged and disability services. Previous research has highlighted significant challenges with establishing reliable methods to collect care outcome data for people ageing with cognitive and communication impairments. For example, the cognitive impairments inherent with dementia present challenges for recruitment,^15^ managing consent^16^ and engagement in research processes.^16^, ^17^ Impairments can also make results difficult to interpret due to vagueness in speech, decreased vocabulary, poor reasoning of verbal information, confabulations or ‘pseudo‐reminiscences’, perseverations, and confused word associations.^18^, ^19^

There is, however, a growing recognition that, with the right support, people with dementia are capable of expressing their views, needs and concerns.^20^, ^21^ It is recognized that understanding the experiences of people with dementia is important for evidence‐based programme and service delivery.^17^, ^22^, ^23^ This is especially so in a CDC aged care market where the success of programmes must be understood through the rubric of meeting peoples' preferences and needs.

There is a need for methodological innovation to support the development of valid, accessible and reliable assessment approaches that promote the involvement of older populations living with cognitive and communication impairments—who are the key audiences for these programmes. For people with dementia, new tools have been developed to support assessment of the health‐related quality of life, which have been designed to capture the perceptions of people with dementia as they relate to their physical, mental, social and health status.^24^ However, for high users of care services in the home or for those who live in residential aged care services, there is also a need to capture people's views about the aspects of their quality of life most impacted by care services. This is called *social care‐related quality of life* (SCRQoL).^25^

### 1.1 Assessment of ScRQoL

The Adult Social Care Outcomes Toolkit (ASCOT) is a valid and reliable measure focused on areas of quality of life that can be attributed to care services—SCRQoL.^25^ The eight domains cover the core or lower order domains, including personal cleanliness and comfort, accommodation cleanliness and comfort, food and drink, feeling safe and also higher‐order domains, including social participation, occupation and control over daily life. The eighth domain, dignity, asks respondents to consider how treatment by care staff makes them feel.^25^ These domains are directly relevant to the goals of the CDC Home Care Package (HCP) in Australia, the country in which this study was conducted.^4^

For each domain of ASCOT, there is one item with four response options, relating to four conceptual outcome states (ideal state, no unmet needs, some unmet needs and high unmet needs). ASCOT has been used extensively to assess care‐related outcomes in community‐dwelling service using populations in the United Kingdom,^26^, ^27^, ^28^ Europe^29^, ^30^, ^31^ and more recently in the Australian older population.^32^, ^33^, ^34^, ^35^ Despite its high validity and reliability,^36^, ^37^, ^38^ barriers exist to engaging sections of the aged and disabled cohort to report on their own outcomes.^39^ As such, it is important to better understand the value of utilizing different formats and degrees of support to better represent the direct perspective of clients with cognitive and communication impairments.

This study outlines the value of alternative methods to promote involvement in the assessment of care‐related outcomes that is inclusive of the voice of the most vulnerable service users. In this study, we trialled the use of two alternative ASCOT questionnaire formats within a cascading inclusive methodology that offered assistance and support as it was required. The aim was to understand what supports older service users with varying degrees of cognitive and physical impairments needed to participate in reporting their own care‐related outcomes, and the value of this reporting to illuminate the service use experiences of this vulnerable cohort.

## 2 METHODS

This study was conducted from June 2017 to February 2018, and explored the usefulness of two ASCOT questionnaire formats and various other forms of support ‘as needed’ to promote self‐reporting of care‐related quality of life outcomes.

Research questions included:

1. Does the use of alternative questionnaire formats enable direct reports of outcomes from service users who would usually rely on proxy reporting (e.g., those with greater cognitive or physical impairments)?
2. What other types of support do participants require for each of the two different questionnaire formats?
3. Are the findings useful to inform understandings of care‐related quality of life for community‐dwelling people with complex needs receiving supports?
4. Are some service users unable to be supported to report against their own outcomes using the proposed methods?

**Figure 1:**
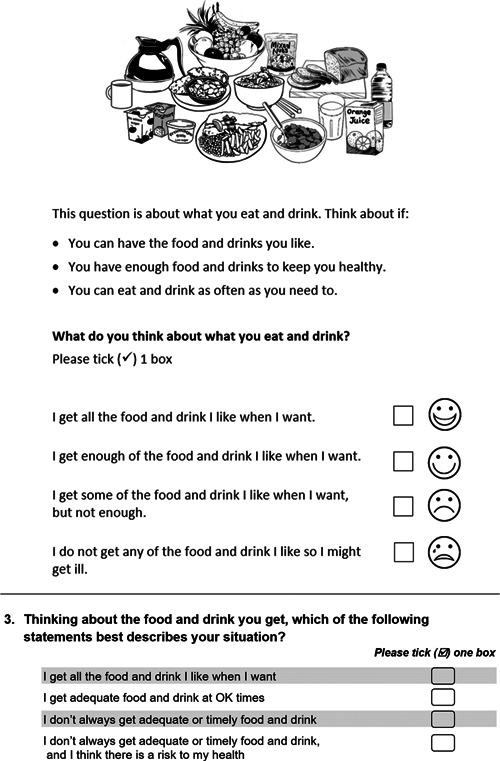
ASCOT ‘Food and Drink’ domain, ASCOT‐ER Pictures, Stem and Response options (top), SCT4 version (below). ASCOT, Adult Social Care Outcomes Toolkit; ASCOT‐ER, ASCOT‐Easy Read. Source: ©University of Kent. Reproduced with permission. All rights reserved.

This study explored the utility of two versions of ASCOT to promote the inclusion of a greater diversity of service users to self‐report their current SCRQoL. The ASCOT SCT4 is a standard, self‐complete questionnaire, designed to assess current SCRQoL in the context of receiving care‐related services with four response options.^25^ The ASCOT‐Easy Read (ASCOT ER) is a modified version of the ASCOT SCT4, initially developed for use with people with autism and intellectual disability,^40^ and later adapted for use with older people with cognitive impairment.^41^ The Easy Read format uses black and white illustrations and plain text to convey the meaning of each of the ASCOT SCRQoL domains. The selection of one of four response options is supported by a visual scale and text‐based response categories. See Figure 1 for an example comparing the ASCOT SCT4 vs ASCOT ER 4 level format.

### 2.1 Recruitment and procedure

The study was conducted in collaboration with a service provider for community‐dwelling older people with complex needs through the Australian government‐funded HCP Programme.^42^ Inclusion criteria were current receipt of HCP supports by this service provider and being resident in the Illawarra‐Shoalhaven region of NSW (Australia). The service provider had been previously involved in the adaption of the ASCOT‐ER tool for use in the older population^41^ and was keen to understand to what extent the ASCOT‐ER would enable the greater voice of their clients reporting on their care‐related outcomes.

The service provider made initial contact with *n* = 299 potentially eligible participants through a mailed out research information pack. The pack included a letter from the provider encouraging participation in the research. It contained a plain language information flyer that outlined the aims of the research and what would be involved for those who were willing to take part. Potential participants were advised, if they consented to take part, a researcher would make arrangements to come to their home to discuss their experiences and test two different questionnaire formats. The flyer included a photograph of the lead researcher to create a personal connection with potential participants, and invited them to make contact via phone, email or mail if they were willing to participate.

In response, 63 (*n* = 63) potential participants (or their carers) made contact by phone or email. The first author answered their questions and provided more information about the study. This included advising them that they would be sent a self‐complete questionnaire (the ASCOT SCT4), but if they could not complete it on their own they could do it with support when they met with the researcher face‐to‐face. At this point in the process, 14 (*n* = 14) participants either declined or were deemed ineligible to take part due to not meeting the inclusion criteria. One participant, whose carer had initiated the phone contact, was deemed unsuitable for participation due to their advanced dementia and was identified at this point as needing alternative methods to assess their outcomes. Appointments were made with 48 (*n* = 48) participants.

#### 2.1.1 Ethics approval and consent process

Approval for the conduct of the study was provided by the University Human Research Ethics committee (HREC Approval 16/236) and addressed the concerns relevant to the ethical conduct of research with people with dementia.^43^ Participants were mailed an ASCOT SCT4, an additional plain language Research Information Sheet and a Consent Form.

On commencement of the research interview, all participants were supported to read through the information, ask any questions and discuss their understanding with the researcher. In any cases where participants showed any confusion, written proxy consent of a care partner was also obtained. During the interviews, all participants were also observed to monitor process assent through their willingness and interest to discuss and answer the questions.^16^

Demographic, functional and cognitive data was then collected directly from participants in a supported interview format. Ths which included age, language spoken other than English, gender, carer status, carer coresidence, education level, self‐reported diagnosis of dementia, monthly family finances and level of HCP. Cognitive status was screened using the Mini‐Cog© (a score of <3 was used as an indication of cognitive impairment).^44^, ^45^ General functional ability was assessed using the Home and Community Care (HACC) functional screener. This tool has a maximum total score of 16, with a lower score indicating more difficulty managing daily activities of living.^46^, ^47^

#### 2.1.2 The cascading methodology

The interviews conducted within this study acted as both a way of collecting data about the experience of completing either version of the ASCOT questionnaire and, providing support and assistance to those who needed it to ensure their participation in the study was ‘manageable’. Whether or not the participants were able to self‐complete the ASCOT SCT4 directed the way the method cascaded during the research interview. If participants were able to independently complete the ASCOT SCT4, they were subsequently interviewed about their responses. If assistance to complete the ASCOT SCT4 was required on the basis of visual or physical impairment, this was provided by the researcher. However, if assistance was required due to the comprehensibility of the questionnaire, they were instead provided with the Easy Read format (ASCOT‐ER). Assistance was again provided on an ‘as needed’ basis, but this time included support for comprehensibility, as well as to aid the focus and/or orientation of participants to promote complete responses for each domain if required.

Detailed field notes were also taken by the researcher regarding the types of assistance and support required by all participants to complete the ASCOT SCT4 or ASCOT‐ER questionnaires.

### 2.2 Data analysis

All interviews were audio‐recorded and transcribed. Transcripts and field notes were placed in NVivo 11 and deductively analysed, using the framework for research cohesiveness to identify the supports that were required to support a manageable, meaningful and comprehensible assessment experience for the participants.^41^ Participants were classified as either being ‘independent’ or ‘assisted’ for three parts in the research process: 1. Replying (as part of the recruitment process) 2. Providing consent and 3. Completing the ASCOT questionnaire. This was further analysed to identify whether the assistance was required to ‘manage’ the demands of the process (e.g., holding a pen, writing or talking on the phone, maintaining focus on topic, prompting to complete responses for each domain) or related to ‘comprehensibility’ (e.g., ability to make sense of the domain through the text explanation or the pictorial).

#### 2.2.1 Statistical analysis

All demographic, functional and cognitive data collected were entered into a spreadsheet and analysed using Statistical Package for Social Sciences (SPSS 24) (SPSS, 2016). Univariate analysis was conducted on demographic and functional data to identify if there were significant differences (*p* < .05) between the characteristics of those able to complete the ASCOT SCT4 vs the ASCOT‐ER formats. Tests for normality were conducted to confirm the homogeneity of variance (SPSS 2016). With regard to the continuous variables, only age (in years) was normally distributed—and this was analysed using a *t*‐test. All other continuous variables (e.g., HACC and Mini‐Cog) utilized Kruskal–Wallis. Tests of normality of categorical variables revealed a lack of homogeneity for all variables. As such, Fisher's exact test was utilized to analyse statistical differences between all categorical variables in the different groups.

#### 2.2.2 Calculating current SCRQoL

Participant responses were recorded during the interview and then manually entered into an ASCOT Excel Spreadsheet for each outcome state per domain. The excel spreadsheets convert raw scores (1 = Ideal state; 2 = No needs; 3 = Some needs; 4 = High needs; −9 = No response) into weighted scores, which reflect UK population preferences (Current SCRQoL = [0.203 × weighted score] − 0.46]) as Australian preferences do not yet exist. The spreadsheets use an algorithm to calculate an overall score ranging from −0.17 to 1.00.^28^ Scores of one represent the ‘ideal’ outcomes across all eight domains and a score of zero represents a state of quality of life that is equivalent, according to the general population, to ‘being dead’. Negative scores indicate a state worse than death.^10^

The ASCOT‐ER differs from other questionnaires in the toolkit by asking two separate questions in the ‘Safety’ domain. This allows participants to distinguish between safety in the home and that experienced in the local area. To enable the calculation of an overall current SCRQoL score using the weighted preferences, and comparability with the other ASCOT measures, in this study, the ‘Safety’ score indicating the highest unmet need for each participant was utilized. Further information about the ASCOT tools, resources and scoring can be found online.^48^

## 3 RESULTS

Overall, there were 63/299 responses to the mailed research invitation (21% response rate). However, *n* = 15/63 of these chose not to take part in the study. A total of 14/15 of these responses were family carers advising ineligibility (e.g., due to the participant being in hospital or admitted to residential care). One other response, also from a carer, confirmed that the HCP recipient was immobile and nonverbal. As such, this participant was identified as needing alternative methods to assess their outcomes.

In total, 48 (*n* = 48) respondents agreed to participate. Five did not complete the study requirements due to sickness (*n* = 1), unwillingness to trial alternative research methods (*n* = 2) or identification of the need for other assessment methods during the research trial (*n* = 2).

### 3.1 Demographic characteristics of participants

Overall, demographic, functional and current SCRQoL data were obtained from *n* = 43 participants. The demographic characteristics of those able to rate their current SCRQoL through completion of either the ASCOT SCT4 or the ASCOT‐ER are displayed in Table 1. Participants in the study were mostly: old (mean age: 84.2 years), female (69%), supported by a family carer (67%; 23% coresident), had more than a high‐school education (53%) and reported adequate financial circumstances (usually had some money left over at the end of each month) (65%). People who spoke a language other than English were not well represented and less than 25% of respondents were receiving the highest level of care package (Level 4).

**Table 1:** Characteristics of participants using ASCOT SCT4 versus ER

Demographics	Total (*n* = 43)	SCT4 (*n* = 35)	ER (*n* = 8)	*p* Value
Age, mean (SD)	84.27 (7.1)	83.56 (7.2)	87.38 (6.17)	.000*
Gender, female *n* (%)	28 (65%)	24 (69%)	4 (50%)	.121
LOTE, yes *n* (%)	1 (2%)	1 (3%)	0 (0%)	1.000
Carer, yes, *n* (%)	29 (67%)	22 (63%)	7 (88%)	.099
Coresident, yes, *n* (%)	10 (23%)	8 (12%)	2 (29%)	.166
Dementia, yes, *n* (%)	3 (7%)	1 (3%)	2 (25)	.032
Education, >highschool, *n* (%)	24 (56%)	21 (60%)	3 (37.5)	.571
Finances, some left over, *n* (%)	28 (65%)	22 (63%)	6 (75)	.229
Mini‐cog, mean (SD)	4.03 (1.44)	4.55 (0.88)	2 (1.41)	<.001*
HACC, mean (SD)	11.79(2.74)	23.38 (2.24)	9.38 (3.07)	.018*
Package level, *n* (%)				
1	1 (2%)	1	0	–
2	27 (63%)	21	6	–
3	5 (12%)	4	1	–
4	10 (23%)	9	1	–

Abbreviations: ASCOT, Adult Social Care Outcomes Toolkit; HACC, Home and Community Care; LOTE, language other than English. *Statistically significant difference.

### 3.2 Value of support to make assessment more manageable for all service users

Overall, 35/48 participants made an attempt to complete the ASCOT‐SC4 self‐complete survey. Of those, 24/35 were able to complete the SC4 independently. For the other 11/35, participants requested physical assistance due to functional impairment (e.g., visual, physical, fatigue). Others also requested support due to a reported lack of confidence, or a desire to clarify the meaning of some questions before choosing a response.

For those 12/48 who were unable or unwilling to attempt completion of the ASCOT‐SCT4, 8/12 were successfully assisted by the researcher to complete the Easy Read format, 2/8 were unable to complete even with assistance and 2/8 declined to make an attempt even with support.

For those eight participants who were aided by the Easy Read format, success was associated with the researcher providing support for orientation, focus, comprehension and completion of the questionnaire. This support was mostly initiated by the researcher on the basis of an assessed need. Rarely was this support directly requested by the participants.

Finally, there were three participants who were unable to be supported to use the Easy Read format. One participant was identified when their carer replied to the research invitation, and two others were unable to be successfully supported during the trial.

See Table 2 for details of needs for assistance and support for successful completion during the various stages of the research and assessment process.

**Table 2:** Need for assistance/support for recruitment and data collection

Tool	Recruitment	Consent	Completion	Comments
SCT4	10/35	0/35	11/35	Assistance mainly to promote ‘Manageability’ of the processes e.g., visual, hearing and writing. Also, some due to anxiety, confidence, loss of interest or fatigue.
ER	4/8	3/8	8/8	Assistance mainly to promote ‘Comprehensibility’ e.g., support for orientation, focus, comprehension and meaning‐making.

### 3.3 Value of easy read format to make assessment more comprehensible

**Figure 2:**
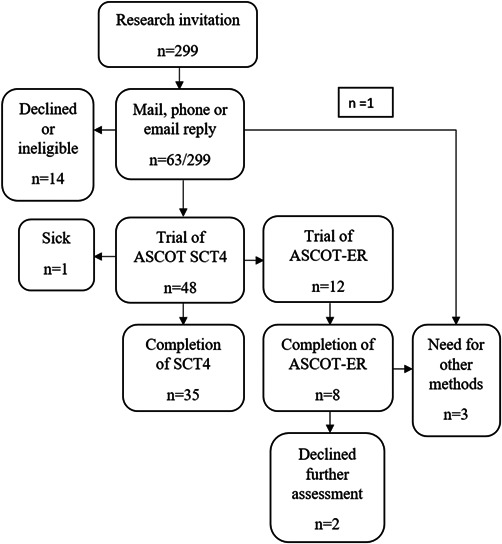
Study flowchart. ASCOT, Adult Social Care Outcomes Toolkit; ASCOT‐ER, ASCOT‐Easy Read

The flowchart in Figure 2 of the cascading inclusive methodology shows how it allowed for more people with cognitive impairment to complete the questionnaire than if the study relied only on the standard questionnaire format (ASCOT SCT4).

Statistical analysis showed significant differences in the profile of those who were able to be supported to complete the ASCOT SCT4 versus the ASCOT‐ER. That is, those who could not complete the ASCOT SCT4 but were successfully supported to complete the ER format to rate their current SCRQoL were older (*p* < .000) and also more cognitively (*p* < .001) and functionally impaired (*p* = .018).

While the size of the study prohibited statistical analysis of differences, comparisons of descriptive data indicate there were also some differences in the self‐reporting of met and unmet needs across the eight domains for the two groups (ASCOT SCT4 vs. ASCOT‐ER). For the ASCOT‐ER group, scores in seven of the eight domains, and their overall current SCRQoL scores, were lower than those who were able to complete the ASCOT SCT4 (see Table 3 for details). The two domains where self‐reported needs were higher for the ASCOT‐ER group than for the ASCOT SCT4 group were ‘Personal cleanliness and comfort’ and ‘Social participation’. The exception was for ‘dignity in care’, with the ER group indicating more ‘ideal’ and ‘no needs’ states than the ASCOT SCT4 group that is those with more cognitive and functional impairment rated the kindness and respect with which they were treated by care staff as in a mostly ‘ideal state’. For both groups, self‐reported needs in the ‘social’ and ‘occupational’ domains were the highest.

**Table 3:** Comparisons of current SCRQoL for participants (ASCOT SCT4 vs. ASCOT‐ER)

Average SCRQoL % of the total	SCT4	ER	Difference
Accommodation	91.43	83.33	8.1
Personal cleanliness	93.33	70.83	22.5
Food & drink	97.14	87.5	9.64
Safety	81.9	75	6.9
Social	73.33	62.5	10.83
Occupation	68.57	62.5	6.07
Control	77.14	75	2.14
Dignity	88.57	100	−11.43
**Overall SCRQoL (%)**	**0.86**	**0.79**	**0.07**

Abbreviations: ASCOT, Adult Social Care Outcomes Toolkit; ASCOT‐ER, ASCOT‐Easy Read; SCRQoL, social care‐related quality of life.

## 4 DISCUSSION

There is a growing emphasis on supporting ageing in place through the provision of long‐term care in community settings through CDC models. To evaluate the outcomes associated with policy change, there is a need for valid and reliable assessments which are inclusive of the voice of all service users. However, this presents numerous challenges when programmes are supporting vulnerable populations with multiple comorbidities, including those ageing with cognitive and communication impairments.

This study specifically explored the value of a cascading inclusive methodology, where increasing supports were introduced as needed by participants as a way of promoting the cohesiveness (comprehensibility, manageability and meaning) of assessment of SCRQoL. Specifically, it explored the value of two alternative formats of the ASCOT (ASCOT SCT4 and ASCOT‐ER) and degrees of functional support needed to assess SCRQoL in older people with complex needs who were recipients of an HCP in a region of NSW, Australia.

### 4.1 Easy read formats promote comprehensible and meaningful assessment

Overall, the use of an Easy Read format (ASCOT‐ER) contributed to supporting people with greater levels of cognitive and functional impairment to rate their current SCRQoL. The pictorials used within the questionnaire supported the comprehensibility and meaningfulness of the questionnaire—promoting greater engagement and meaningful self‐reflection on care domains. This is consistent with the results from a study that used a cognitive interviewing protocol to adapt the questionnaire for use within this cohort.^41^ It is also consistent with other research, which has highlighted the value of visual methods and aids within research with people with dementia to promote their engagement.^49^ The use of ER principles is mandatory in the NHS for all health information resources, to ensure that all service users have access to health information in an accessible form.^50^ Results from this study suggest the benefits of tailored ER in the design of evaluation tools to promote the inclusion of the voice of older people living in community settings with cognitive and communication impairments. Given they are a primary target audience for programmes, these types of formats should be seen as mandatory, rather than as exceptional, in the evaluation of home‐based support programmes.

### 4.2 Additional supports needed to promote more manageable assessment

In this study, the successful completion of the ASCOT questionnaires in both formats (ASCOT SCT4 or ER) benefitted from the support of the interviewer. The cascading inclusive methodology provided value to participants who needed both physical and/or cognitive support. In addition, unlike other inclusive methods that propose a one‐sized fit all adjustment (such as an Easy Read format for all participants), the cascading inclusive methodology adjusts according to need. For example, almost a third who completed the standard format (ASCOT SCT4) also benefitted from assistance to make their participation more ‘manageable’. This was mostly to support physical impairments (e.g., vision, physical limitations, fatigue) though some also reported a lack of confidence, or a desire to clarify the meaning of some questions before choosing a response. In contrast, for those requiring the ER format, assistance was required by *all* participants and was frequently more intensive, including support to maintain orientation, focus, comprehension and completion of the questionnaire.

The need to adapt methods and provide support for people with dementia to participate in research is established.^51^, ^52^ Adaptation is useful, specifically for older people with cognitive impairment to promote meaningfulness in the context of their life stage.^41^ This study reinforces that the ER format was useful to improve ‘comprehensibility’ of the domains, the use of a supported interview protocol was also essential to ensure engagement with the questionnaire was ‘manageable’.^41^ While preliminary, the early findings from this study show that support is necessary for use of the Easy Read version of the questionnaire with this cohort.

### 4.3 Insights gained through a cohesive methodology

Despite the challenges, the use of multiple questionnaire formats and a cascading inclusive methodology was useful to highlight better self‐rated outcomes in lower order domains and more needs in higher‐order domains for all home care users. This is similar to results from other studies using ASCOT, including the ACCOM study in Australia^53^ and also from studies of long‐term care users in the community in the United Kingdom.^10^

Due to the utility of the ASCOT‐ER administered in a supported interview format, this study is the first to collect self‐reported care‐related quality of life outcomes in community‐dwelling older people with cognitive impairment. This group would either normally be excluded for service evaluations, or have a proxy respond on their behalf. It is important to note that these participants reported consistently poorer outcomes across seven out of eight domains. They also reported better outcomes for the eighth domain—‘dignity in care’. This could suggest this cohort may experience different benefits from their supports or could reflect the value of different types and intensities of care that people with higher needs were receiving. While only a small scale study, these results merit further investigation to maximize insight into the value of aged care support programmes for this vulnerable cohort.

### 4.4 Limitations and future research

This study was conducted for the purposes of understanding the methods that may be required to increase the involvement of vulnerable older people in reporting outcomes from their aged care services. It was conducted in a small sample of home care users from a service provider in a single geographical region in Australia. The study was very time intensive and the researchers required dedicated training to provide the needed support. This may limit the future transferability of some of these methods beyond the research environment. Future research is therefore required to apply these innovative methods to assess outcomes in a representative sample of service providers and users in different geographical locations. The results obtained about users SCRQoL may not be representative of the population of the provider, the region or the broader home care population. However, this was not the aim of the study.

We had limited success in assessing the outcomes of people with more moderate or advanced dementia. This suggests there is still a further need for methodological innovation to promote their inclusion in service evaluation. One approach that may have merit is the multi‐methods ASCOT CH4^54^—which has been used to promote greater inclusion of the voice of older people with cognitive and communication impairment living in institutional/care home settings but has not yet been feasible in a community setting.

The use of a mail out invitation to take part in the research was also not useful in supporting participation in home care users who speak languages other than English, those without the support of an informal carer and those with more moderate and advanced dementia. As such, alternative methods for recruitment are needed to overcome the challenges of recruiting people with dementia for service evaluation research, even when using multiple methods.^15^

Future research should focus on supporting larger‐scale research in a representative population. It should also consider the value of training service providers to adapt cascading supportive approaches to support their use of assessment tools as part of routine care planning and service evaluation. Finally, further methodological innovation is needed to ensure that the preferences and needs of those living in community settings with more moderate and advanced impairments are understood and met.

## 5 CONCLUSION

Overall, this study highlights the value of a cascading inclusive methodology that has methods and supports that adjust to the cognitive and physical needs of community‐dwelling older people with complex needs. Offering people with cognitive and physical impairments a range of methods and supports to meet their needs allowed us to include the direct perspective of vulnerable people in reporting on their own needs. This is crucial if we are to evaluate the success of programmes and policy changes that aim to support older people living with cognitive and/or communication impairments as one of their primary target groups.

## CONFLICTS OF INTEREST

The authors declare that there are no conflicts of interest.

## AUTHOR CONTRIBUTIONS

Lyn Phillipson was the chief investigator on the study and was responsible for the overall study design, conduct, data collection, data analysis and led the writing of the manuscript. James Caiels and Ann‐Marie Towers contributed to the study design, provided advice on data analysis and contributed to drafts of the manuscript. Louisa Smith was involved in supporting data analysis and was a major contributor in writing the manuscript. All authors read and approved the final manuscript.

## Data Availability

Data are not available due to the conditions of the ethics approval.
